# The Chloride Channel Regulator, Calcium-Activated-1 Is Expressed in Synoviocytes and Articular Chondrocytes in Health and Disease

**DOI:** 10.1369/00221554261423720

**Published:** 2026-03-15

**Authors:** Judith Bushe, Jenny Fürstenau, Florian Bartenschlager, Simon Dökel, Vladimir M. Jovanovic, Gundula Rösch, Zsuzsa Jenei-Lanzl, Frank Zaucke, Kristina Dietert, Achim D. Gruber

**Affiliations:** Institute of Veterinary Pathology, Freie Universität Berlin, Berlin, Germany; Institute of Veterinary Pathology, Freie Universität Berlin, Berlin, Germany; Institute of Veterinary Pathology, Freie Universität Berlin, Berlin, Germany; Institute of Veterinary Pathology, Freie Universität Berlin, Berlin, Germany; Bioinformatics Solution Center, Freie Universität Berlin, Berlin, Germany; Department Trauma Surgery and Orthopedics, University Hospital Frankfurt, Goethe University Frankfurt/Main, Frankfurt, Germany; Department Trauma Surgery and Orthopedics, University Hospital Frankfurt, Goethe University Frankfurt/Main, Frankfurt, Germany; Division for Biochemistry of Joint and Connective Tissue Diseases, Department of Orthopedics, University of Ulm, Ulm, Germany; Department Trauma Surgery and Orthopedics, University Hospital Frankfurt, Goethe University Frankfurt/Main, Frankfurt, Germany; Veterinary Centre for Resistance Research, Freie Universität Berlin, Berlin, Germany; Institute of Veterinary Pathology, Freie Universität Berlin, Berlin, Germany

**Keywords:** arthritis, cartilage, CLCA1, synovial membrane

## Abstract

The chloride channel regulator, calcium-activated-1, CLCA1, acts as a multifunctional secreted glycoprotein in anion channel modulation, mucus homeostasis, immune regulation, and other functions. So far, it has been described in goblet and other mucus-producing cells in mucous membranes, primarily of the respiratory, alimentary, and urogenital tracts. Here, we identify the expression of CLCA1 in fibroblast-like synoviocytes and superficial as well as, to a lesser extent, intermediate zone chondrocytes of diarthrodial articular joints. The expression pattern was found to be conserved in major joints in mice, pigs, and humans. First analyses of degenerate or infectious inflammatory joint conditions in mice or pigs, respectively, suggest rather continuous expression levels in articular disease. We speculate that CLCA1 may be involved in articular anion conductance, proteoglycan homeostasis, and inflammation of articular joints, possibly similar to analogous functions of this molecule as established in mucous membranes of the lungs and intestine:

## Introduction

Since its discovery in 1998, the chloride channel regulator, calcium-activated-1 (CLCA1), previously also termed chloride channel accessory 1, mCLCA3, or gob-5 in mice, has exclusively been described in mucin-producing cells and glands of the alimentary, respiratory, and reproductive tracts as well as the skin in humans, mice, pigs, horses, and other mammals.^1–5^ The structure of this secreted glycoprotein appears to be highly conserved across mammals. An approximately 120 kDa precursor protein is cleaved to produce an approximately 85 kDa amino (N)-terminal and a 35 kDa carboxy (C)-terminal subunit, both possessing several N-linked glycosylation sites. The amino terminal fragment contains a zinc metalloprotease domain with a conserved HExxE catalytic motif which is capable of self-cleavage of the primary translation product.^6^ The two subunits remain associated with each other, likely by disulfide bonds, and are fully released via secretory granules by mucinous cells into the mucus where CLCA1 can consistently be detected in large abundance.^3,5,7–11^

There is ample experimental evidence that CLCA1 acts as a multifunctional protein, but its primary role and how the different proposed functions are connected is poorly understood. In contrast to its originally suggested function as a bona fide channel protein, it is now thought to be indirectly involved in mucus hydration by modulating select other anion channels, including anoctamin-1 (ANO1, also known as TMEM16A), a voltage-gated calcium-activated channel for chloride and bicarbonate.^12–15^ In addition, CLCA1 can also process N-terminal mucin (MUC) 2 in the murine colon by proteolysis and formation of non-covalent oligomers, thereby contributing to mucin homeostasis.^16^ Recent structural analyses have established that it forms large oligomeric complexes with some resemblance to the metzincin metalloproteinases, supporting CLCA1’s role in mucus remodeling by cleaving glycosylated substrates.^17^ Moreover, it was found to regulate mucus cell differentiation in the airways by inducing mucus gene transcription via downstream mitogenic-activated protein kinase (MAPK) 13/p38δ MAPK signaling pathway.^18^ CLCA1-deficient pigs fail to produce MUC5AC-expressing mucous cells throughout the lung and the intestine.^19^ CLCA1 was also associated with airway inflammation and immune homeostasis in a *Staphylococcus aureus* pneumonia model in CLCA1-deficient mice. Here, lack of CLCA1 resulted in reduced neutrophilic infiltration into the bronchoalveolar space during bacterial infection as well as lower expression of the pro-inflammatory cytokine IL-17 and the neutrophil attracting chemokine CXCL-1, which is the murine orthologue to human CXCL-8, also known as interleukin (IL)-8.^20^

Several studies have linked CLCA1 to non-infectious inflammatory airway conditions with aberrant function and secretion of mucus, such as chronic obstructive pulmonary disease (COPD), cystic fibrosis (CF), and asthma.^5,11,17,21–24^ Increased amounts were detected in the lungs and bronchoalveolar lavage (BAL) fluid of ovalbumin (OVA)-challenged mice and in the BAL of asthmatic patients.^7^ The same observation was made in the BAL of horses with equine asthma, where it was linked to airway goblet cell hyperplasia.^9^ Anti-CLCA1 antibody treatment remarkably ameliorated the asthmatic phenotype in the OVA-challenged mouse model.^25^ Furthermore, single-nucleotide polymorphisms associated with certain haplotypes of the human CLCA1 gene have been linked to increased susceptibility to asthma, CF, and COPD.^21,26,27^

Moreover, additional roles of CLCA1 have been proposed in human colorectal, pancreatic, hepatocellular, and ovarian cancer, but its significance in tumors is still uncertain.^22,28,29^ Finally, a link of CLCA1 to joint disease and arthritis pain has been proposed. Its mRNA was found drastically increased in the dorsal root ganglia of mice with acute antigen-induced arthritis in their knee joints, and CLCA1-deficient mice had less articular swelling than wild-type mice.^30^ The mechanisms of these effects, however, remained elusive.

Here, we for the first time identified strong expression of CLCA1 in synoviocytes and articular chondrocytes, a tissue microenvironment where this multifunctional molecule has not yet been described. We report on its joint-associated cellular distribution pattern in humans, mice, and pigs and speculate on its possible functions in articular compartments, based on parallels to its presumed role in respiratory and intestinal mucous membranes. Finally, we tested for altered CLCA1 expression levels in various joint pathologies to obtain first evidence toward its possible role in inflammatory and degenerative joint diseases.

## Materials and Methods

### Tissue Samples

Numerous healthy major diarthrodial joints of all four extremities of 12-week-old male and female C57BL/6J mice were analyzed. In addition, knee joints of 8-week-old, 3-, 6-, and 18-month-old male and female C57BL/6J mice were employed for age comparisons (*n*=3–6 per time point). Details of all animals used are listed in Supplementary Table S1.

All mice were anesthetized by intraperitoneal injection of combined ketamine and xylazine, followed by exsanguination via the caudal caval vein. Entire skinned front and hind limbs as well as nasal, tracheal, and auricular cartilage were fixed in 10% formalin for 24 hr. Limbs were decalcified with ethylenediaminetetraacetic acid (EDTA) 20% pH 7.0 at 60C for 2 weeks and processed following standard procedures for formalin-fixed, paraffin-embedded (FFPE) material.

Whole C57BL/6J mouse embryos at stages E12.5, E16.5, and E18.5 as well as whole postnatal day 1 neonates (*n*=3 per time point) were immersion fixed in 10% buffered formalin for 24 hours, followed by sagittal half sectioning and embedding in paraffin. The complete left hind limbs of mice aged 10, 20, or 30 days (*n*=3 per time point) were immersion fixed in 10% buffered formalin, decalcified with 20% EDTA (pH 7.0) at 60C for 2 weeks, embedded in paraffin, and serially sectioned for histology.

For destabilization of the medial meniscus (DMM) experiments, male C57BL/6J mice at 12 weeks of age underwent surgery as previously described.^31^ Pathological outcomes were analyzed histologically at 2, 4, 8, and 12 weeks after surgery (*n*=4–6 per time point) and scored according to previously established Osteoarthritis Research Society International (OARSI) and synovitis scores.^32,33^

Normal porcine synovium samples from all major diarthrodial joints of the extremities of both sides as well as cartilage from the left and right tarsal and knee joints were collected from three healthy pigs, two females and one male, 12–18 weeks of age. These pigs were obtained from a research facility after their finalization for other purposes, where they had been kept under defined experimental housing conditions. In addition, synovium samples were collected from 23 domestic farm pigs suffering from spontaneous joint diseases and routinely necropsied for diagnostic purposes at the Department of Veterinary Pathology, Freie Universität Berlin. These pigs were between 5 weeks and 2 years of age with the majority younger than 6 months. Inclusion criteria were defined as a postmortem interval of less than 24 hours and a minimum weight of 5 kg to ensure comparable amounts of coherent synovial tissue and sufficient cartilage thickness. The spectrum of examined porcine tissue samples varied between animals, depending on their actual individual distribution of joint disease. Tissue samples were processed for histology as described for mice.

Samples from normal equine synovium were obtained from FFPE archival material of horses of 6 weeks to 15 years of age and both genders. The horses underwent diagnostic necropsy procedures for unrelated purposes at the Department of Veterinary Pathology, Freie Universität Berlin, with no evidence of joint disease.

Details of all animal samples used in the present study are summarized in Supplementary Table S1. Human synovium samples were obtained post mortem from tarsal and knee joints of 10 body donor corpses (age ranging from 66 to 97 years, 4 female and 6 male) during educational sessions at the Institute of Anatomy and Cell Biology, Justus-Liebig University, Giessen, Germany (institutional ethics committee approval No. 129/14). Corpses were formalin-fixed for several weeks to months and underwent standard procedures for processing FFPE material.

### Histology, Immunohistochemistry, and Immunofluorescence

FFPE tissues were sectioned at 2- to 4-µm thickness and stained with hematoxylin and eosin (HE). For some aspects of the study, Alcian blue stain (pH 0.5) or the periodic acid-Schiff (PAS) reaction was used. Immunohistochemistry and immunofluorescence were performed as described.^23,34,35^ Briefly, tissue sections were deparaffinized and endogenous peroxidase was blocked using 0.5% H_2_O_2_ in phosphate-buffered saline for 15 min. Either heat or enzymatic antigen retrieval was performed depending on the antibody used. All antibodies were incubated overnight at 4C. The synovium and cartilage of mice were incubated with immunopurified polyclonal rabbit anti-murine CLCA1 antibody α-m3-C-1p diluted at 1:600. Anti-murine CLCA2-antibody αm5-C1-a served as negative control and was used as described.^35^ For pig synovium and cartilage, rabbit anti-porcine CLCA1 immunopurified polyclonal antibody p1-N-1ab-p (1:3000 for chromogen visualization, 1:500 for fluorescent detection) and anti-proteoglycan 4 (also termed lubricin) antibody ab28484 (Abcam, Cambridge, UK; diluted 1:400 or 1:50, respectively) were utilized. Anti-porcine CFTR antibody (pCFTR-C1; diluted 1:1000) served as negative control.^34^ Equine tissues were incubated with rabbit anti-equine CLCA1 antibody (ec8r; 1:400).^9^ Human synovium was incubated with rabbit anti-human CLCA1 antibody EPR12254-88 (ab180851; Abcam) diluted at 1:75. Irrelevant rabbit antibodies (rabbit super sensitive negative control; Biogenex, Fremont, CA) were used at the same dilution as negative control on equine and human tissues.

Additional tests for specificity of antibody binding were performed using pre-absorption of anti-murine and anti-porcine CLCA1 antibodies with the peptides used for their immunization or an unrelated peptide of otherwise identical properties as described.^34^ Secondary biotinylated goat anti-rabbit antibody was applied followed by ABC solution (Vectastain Elite ABC Kit; Vector Laboratories Inc., CA) and incubation with the chromogen 3,3′-diaminobenzidine. Sections were counterstained with hematoxylin.

For immunofluorescent localization of CLCA1 and proteoglycan 4 on serial sections of porcine synovium (primary antibodies as above), Alexa Fluor 488–conjugated and Alexa Fluor 546–conjugated goat anti-rabbit secondary antibodies were used, respectively. Sections were counterstained with Roti-Mount FluorCare DAPI (Carl Roth, Karlsruhe, Germany) which also served as mounting medium.

Immunofluorescent co-localization of CLCA1 and CD68 on FFPE and decalcified mouse metatarsal joints was performed using antibody α-m3-C-1p, diluted at 1:60, and anti-CD68 monoclonal antibody FA-11 (Abcam), diluted at 1:100, respectively. Alexa Fluor 546–conjugated or Alexa Fluor 488–conjugated secondary antibodies (Thermo Fisher Scientific, Darmstadt, Germany) diluted 1:200 were used for separate visualization of anti-CLCA1 and anti-CD68 antibody binding, respectively. Irrelevant rabbit antibodies (Biogenex) at the same dilution as the primary antibodies, respectively, were used as negative control. Sections were counterstained with Roti-Mount FluorCare DAPI (Carl Roth) which also served as mounting medium. Images were taken using a Leica DMi8 fluorescent microscope.

### RNA In Situ Hybridization

Tissue mRNA was hybridized on formalin-fixed, deparaffinized 4-µm-thick sections using the ViewRNA Tissue Assay Core Kit as well as the additional ViewRNA Tissue Assay Blue Module (Invitrogen, Thermo Fisher Scientific). Sections were heat pretreated for 10 min at 90C–95C, followed by protease digestion in a humidified hybridization system for 20 min at 40C. Specific probes were incubated for 2 hr at 40C, diluted in probe set diluent. A probe for porcine EF1α served as positive control on consecutive sections, whereas the probe set diluent without probe (“no probe” control) served as indicator for possible background staining unrelated to probe hybridization. Probes were designed and synthesized by Affymetrix (Sus scrofa EF1α; Catalog-nr. VF1-4295820; Sus scrofa PRG4, Catalog-nr. VPDJXGD; Sus scrofa CLCA1, Catalog-nr. VF1-4295117). Nuclei were counterstained with Roti-Mount FluorCare DAPI (Carl Roth) which also served as mounting medium. Fast Red signals were detected using an Olympus DP 80 microscope (Olympus Co., Tokyo, Japan) equipped with UV light at 600× magnification and 550 nm, whereas the Fast blue signal was detected under bright field illumination.

### Digital Image Analysis

Immunohistochemically stained synovium sections from porcine joint disease samples were scanned for digital image analysis with the Aperio CS2 slide scanner (Leica Biosystems Imaging Inc., Vista, CA) at 400× magnification. Five areas of 200-µm length each were randomly selected throughout the whole synovial lining, resulting in a total annotated length of 1 mm per tissue sample. The corresponding synovial lining was annotated, stained synovial lining cells were counted manually, and the annotated area was analyzed using the trained Aperio Pixel count v9 Algorithm (Leica Biosystems Imaging Inc.). Samples from articular cartilage of these animals were unavailable.

### Data Mining

The NCBI Bioproject (https://www.ncbi.nlm.nih.gov/bioproject) and Sequence Read Archive databases (https://www.ncbi.nlm.nih.gov/sra) were searched for sequencing experiments on porcine models of osteoarthritis. The search yielded datasets of three projects (accession numbers PRJEB8085/ERP009122, PRJEB22000/ERP024317, and PRJEB25953/ERP107920) appropriate for further analysis. The raw reads from these experiments were quickly checked; the bases with low quality of base calling and adapters were trimmed from reads using *cutadapt* 2.10.^36^ The reads were then mapped to the reference pig genome (Sscrofa11.1) with *hisat2* 2.1.0,^37^ assigned to the genes with *featureCounts* 1.6.4,^38^ and the read counts across all samples were normalized within package *DESeq2* 1.28.1 in R.^39^ The normalized counts were then used in statistical analyses.

### Statistics

Statistical tests were performed using the Kruskal-Wallis test followed by Dunn’s post hoc test with Holm’s *p*-value adjustment for multiple comparisons. Illustrations were performed using GraphPad PRISM 9 and 10 (GraphPad Software Inc, La Jolla, CA). *P*-values are depicted as *p<0.05; **p<0.01; ***p<0.001; ****p<0.0001.

## Results

### CLCA1 Is Expressed in the Synovial Intima of Articular Joints Across Mammals

**Figure 1.**
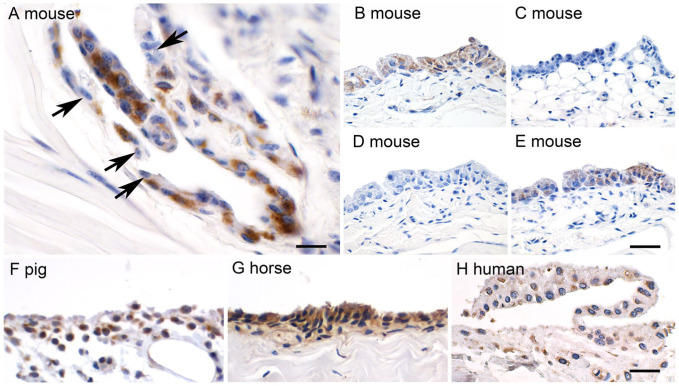
CLCA1 is expressed in synoviocytes of murine, porcine, equine, and human articular joints. (A) Immunohistochemical staining of the metacarpal synovium of an 8-week-old mouse using the anti CLCA1-antibody (α-m3-C-1p), diaminobenzidine as chromogen (brown), and hematoxylin counterstain (blue). The majority of the synovial intima cells stain intensely for CLCA1, whereas a few interspersed cells remain unstained (black arrows). (B) Murine tarsal joint synovium staining for CLCA1 using the same antibody. (C) No staining was observed when a specific antibody (αm5-C1-a) against the related CLCA2 protein was used (for positive control, see Fig. S1). (D) Likewise, pre-incubation of the anti-CLCA1 antibody with the synthetic peptide that had been used for its generation fully abolished the CLCA1 signals. (E) When the anti-CLCA1 antibody was pre-incubated with an unrelated peptide, the signals remained unchanged. (F, G, H) Similar staining patterns were observed when porcine tarsal synovium, equine tarsal synovium, or human tarsal synovium were incubated with species-specific anti-CLCA1 antibodies, respectively. Scale bars (A) = 20 µm, (B–H) = 50 µm.

Strong expression of CLCA1 was detected immunohistochemically in synovial lining cells of all healthy joints from the four limbs of 12-week-old male and female mice (*n*=8), including the hip, knee, tarsal, and metatarsal, as well as shoulder, elbow, carpal, and metacarpal joints. Examples are shown for metacarpal (Fig. 1A) and tarsal (Fig. 1B) joints. Within each joint, we estimate that approximately 80% of the synovial lining cells had strong granular to diffuse cytoplasmic staining with no labeling of apical or basolateral membranes or the nuclei. CLCA1-expressing cells usually clustered in groups of variable cell numbers, intermingled with often more superficial synovial lining cells that lacked CLCA1 expression. No staining was observed in any cell type when antibodies against a related protein of the CLCA family, CLCA2, were used (Fig. 1C). A proper positive control for the localization of the CLCA2 protein on mouse skin tissue is shown in the supplemental material (Fig. S1). To further test for specificity of antibody binding, we pre-incubated both antibodies with the synthetic CLCA1 peptides that had been used to generate these antibodies in rabbits, resulting in complete abolishment of the signals (Fig. 1D). In contrast, when the antibodies were pre-incubated with unrelated peptides at the same concentration, the signals indicative of CLCA1 expression remained unchanged (Fig. 1E).

We then evaluated the CLCA1 expression in several articular joints of other mammals, including pigs, horses, and finally humans, using antibodies that had been specifically generated against their CLCA1 orthologues. In pigs, multiple samples of the synovium from hips, knees, tarsal, and metatarsal, as well as shoulder, elbow, carpal, and metacarpal joints of both limbs of three healthy 12- to 18-week-old animals were tested. In horses, the synovium of 24 healthy major diarthrodial joints of 11 horses from 6 weeks up to 15 years of age of various breeds and gender were included. Sampled synovial membranes originated from the metacarpophalangeal and metatarsophalangeal joints as well as the carpal and shoulder joints. Human synovium samples of the tarsal and knee joints from six male and four female body donor corpses were also investigated. All synovial lining cells had largely similar CLCA1 staining patterns, regardless of the species or anatomic origin, and independent of the areolar, adipose, or fibrous type of synovium tested (Fig. 1F to H).

**Figure 2.**
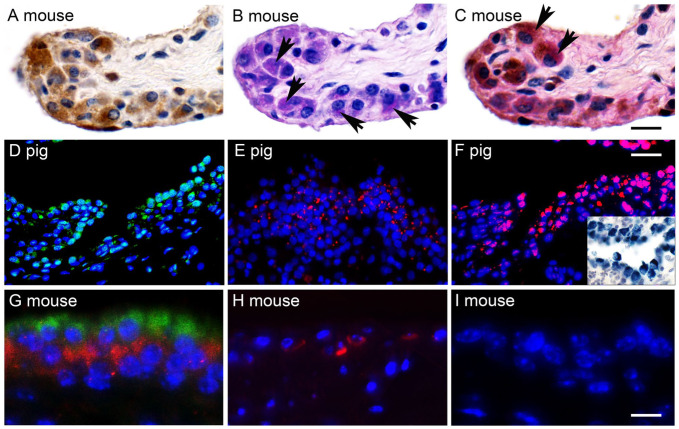
Co-staining of CLCA1-expressing synoviocytes with relevant cell markers. (A–C) Consecutive synovial sections from a murine knee joint were incubated with anti-CLCA1 antibody α-m3-C-1p using diaminobenzidine as chromogen (brown; A), the PAS reaction (B) or both (C), indicating that CLCA1-expressing cells also stained with the PAS reaction (arrows). (D–E) Porcine synovial lining cells that stained for CLCA1 protein (D; immunofluorescence using antibody p1-N-1ab-p and green Alexa Fluor 488 fluorochrome) and mRNA (E; fluorescent in situ hybridization using a porcine CLCA1-specific probe and Fast Red as chromogen) also stained for proteoglycan 4 protein (F; immunofluorescence using antibody ab28484 and red Alexa Fluor 546 fluorochrome) and mRNA (inset; bright field in situ hybridization using a porcine proteoglycan 4–specific probe and Fast blue as chromogen on consecutive sections). (G–H) Immunofluorescence double staining of murine metatarsal synoviocytes (G) and chondrocytes (H) with anti-CLCA1 antibody α-m3-C-1p (G, H; red Alexa Fluor 546 fluorochrome) and anti CD68 antibody FA-11 (G, H; green Alexa Fluor 488 fluorochrome) revealed that distinct subpopulations expressed CLCA1, likely representing type B synoviocytes, or CD68, likely representing type A synoviocytes. Chondrocytes (H) only expressed CLCA1 (red) but not CD68 (green). (I) Consecutive synovium section as in G incubated with irrelevant rabbit antibodies (Biogenex, Fremont, CA) at the same dilution as the primary antibodies, respectively, as negative control. Nuclei were stained with DAPI (blue). Images are merged from three separate excitation-emission channels, 545/25–605/70 nm, 470/40–525/50 nm, and 405/60–470/40 nm. *n*=3 per group. Scale bars (A–C) = 20 µm, (D–F) = 50 µm, (G–I) = 15 µm.

Because CLCA1 is strongly expressed in virtually all respiratory, intestinal, and reproductive mucus-expressing cells where it is thought to be involved in mucus processing and mucus homeostasis,^3,16^ we speculated that CLCA1 function in articular joints may also be associated with mucin-like substances such as proteoglycan 4. To this end, the PAS reaction was performed on murine tarsal synovium to identify cells that express mucopolysaccharides, mucoproteins, and glycoproteins (*n*=3).^40^ PAS positivity was highly correlated with CLCA1-expressing cells (Fig. 2A to C). Furthermore, similar staining patterns were observed in synovial lining cells for proteoglycan 4 protein and mRNA expression as compared with CLCA1 protein and mRNA when immunohistochemistry and RNA in situ hybridization were performed, respectively, on consecutive sections from porcine knee joints (Fig. 2D to F).

We then tested for co-expression of CLCA1 with the type A synovial lining cell marker CD68 using immunofluorescence double staining of murine metatarsal synovium (*n*=3). Green fluorescence revealing expression of CD68 was associated with the cytosol of superficial synoviocytes, consistent with type A synovial lining cells. However, these cells failed to emit red signals, indicating no CLCA1 expression, which was restricted to synoviocytes in deeper layers (Fig. 2G). Co-expression of the two proteins was not observed in any synoviocyte. Adjacent superficial and mid zonal articular cartilage cells expressed only CLCA1 but not CD68 (Fig. 2H).

### CLCA1 Is Expressed in Chondrocytes of Articular Hyaline Cartilage

**Figure 3.**
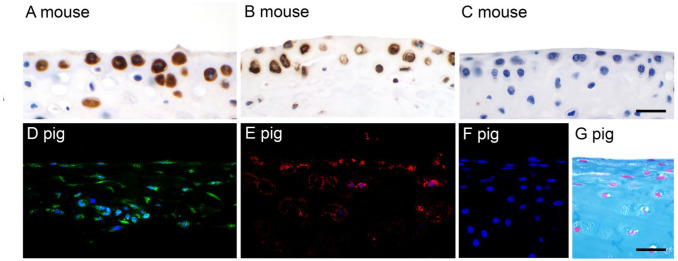
Expression of CLCA1 in superficial and mid zonal chondrocytes of hyaline articular cartilage. (A) Tarsal joint cartilage of a mouse with strong cytosolic labeling, immunohistochemistry using anti-CLCA1 antibody α-m3-C-1p, diaminobenzidine as chromogen (brown), and hematoxylin counterstain (blue). (B) Cells in the same region and with similar numbers stained strongly for proteoglycan 4 expression with antibody ab28484 on serial sections of the same tissue. (C) No staining was observed when an antibody against CLCA2 was used (αm5-C1-a; for positive control, see Fig. S1). (D, E) Similar expression patterns of CLCA1 (D, green fluorescence) and proteoglycan 4 (E, red fluorescence) were detected in chondrocytes of porcine knee cartilage by immunofluorescence using antibodies specifically targeted against the porcine proteins. (F) No signals were observed when a control antibody was used (anti-CFTR pCFTR-C1). Blue, nuclear DAPI counterstain. (G) The same frame was taken for anatomical orientation from an Alcian blue–stained consecutive section. Scale bars (A–C) = 50 µm; (D–G) = 50 µm.

When we further examined the expression pattern of CLCA1 in major diarthrodial articular joints of healthy 8- to 12-week-old mice (*n*=3–6), we also detected strong signals in the cytosol of virtually all superficial and mid zonal chondrocytes of the hyaline cartilage (Fig. 3A). Similar to the synovial lining cells, CLCA1-positive chondrocytes were also found to express proteoglycan 4 as revealed/ by immunohistochemistry (Fig. 3B). Again, an antibody targeting the related CLCA2 protein failed to yield any signals (Fig. 3C; positive control shown in Fig. S1). Similar results were obtained from healthy porcine tarsal and knee joints as well as metacarpal, carpal, and metatarsal joints (Fig. 3D to G). In contrast, we failed to detect CLCA1 expression in chondrocytes of hyaline cartilage from non-articular structures in mice and pigs (*n*=3 per species), including nasal septum, auricular, and tracheal cartilage (Supplementary Fig. S2).

### CLCA1 Protein Expression Is Age-Dependent in Murine Joints

**Figure 4.**
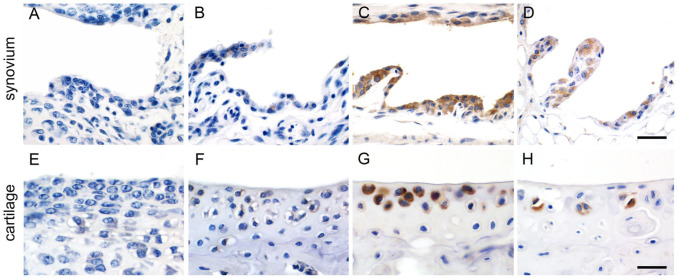
Age-dependent changes in CLCA1 expression in murine tarsal joint synoviocytes (A–D) and articular chondrocytes (E–H; *n*=3 per time point). Immunohistochemical staining with anti-CLCA1 antibody α-m3-C-1p, diaminobenzidine as chromogen (brown), and hematoxylin counterstain (blue). (A) No expression was observed at postnatal day 10 or earlier. (B) First weak expression of CLCA1 was observed on postnatal day 20, along with the formation of a surface lining layer of synoviocytes. (C) Strong and widespread expression of CLCA1 at 8 weeks of age. (D) Weaker signals for CLCA1 in structurally largely unaltered synoviocytes at 18 months of age. (E) No expression of CLCA1 was detected in articular cartilage at postnatal day 10. (F) First weak CLCA1 expression in chondrocytes was observed at 30 days of age, along with the formation of hyaline cartilaginous matrix between the chondrocytes. (G) Strong immunohistochemical signals for CLCA1 at 8 weeks of age. (H) At 18 months of age, weaker signals for CLCA1 in less chondrocytes were associated with age-related structural changes of both chondrocytes and the cartilaginous matrix. Scale bars (A–D) = 40 µm; (E–H) = 25 µm.

Previous data collected from the GEO Profiles database indicated age-dependent changes of CLCA1 expression levels in the small intestine of mice with a strong increase between 8 and 24 days of age (data accessible at NCBI GEO database accession GSE8065).^41^ We therefore tested for age dependence of CLCA1 expression in murine tarsal joints at embryonal stages E12.5, E16.5, E18.5 and postnatal days 1, 10, 20, and 30 (*n*=3 per time point). No signals were detected in synovial lining cells or chondrocytes at any of the embryonal stages investigated as well as postnatal days 1 and 10. First immunohistochemical signals were detectable in 20-day-old mice in superficial synovial lining cells but at no earlier time points (Fig. 4A, B). Morphologically, this correlated with early structural formation of a more distinct synovial lining cell layer at that time. Strongest CLCA1 staining was observed in mice of 8 weeks of age (Fig. 4C), whereas weaker signals in fewer synoviocytes were present in 18-month-old mice, accompanied by age-associated structural changes (Fig. 4D).

Chondrocytes of the murine tarsal joints (*n*=3 per time point) first weakly expressed CLCA1 at postnatal day 30 but not at earlier time points (Fig. 4E, F). Again, strongest staining was detected at 8 weeks of age (Fig. 4G). In 18-month-old mice, overall expression of CLCA1 in tarsal joint cartilage was weaker and many superficial chondrocytes failed to express CLCA1 (Fig. 4H).

To analyze possible sex-specific age-related changes in articular CLCA1 expression, we compared the knee joints of female and male mice aged 3, 6, and 18 months (*n*=3). Both female and male mice had apparently similar reduction of CLCA1 protein staining with increasing age, both in the synovium and cartilage (Supplementary Fig. S3).

### Continuous Synovial Expression of CLCA1 in Health and Disease

**Figure 5.**
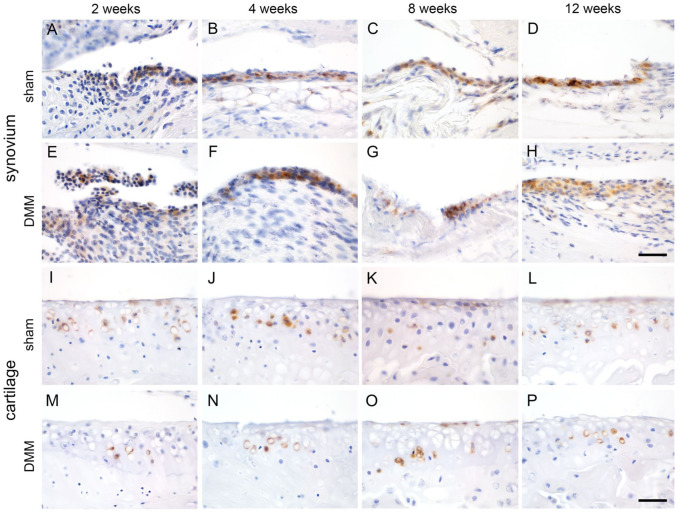
Expression of CLCA1 in articular synoviocytes (A–H) and chondrocytes (I–P) in a murine medial meniscus destabilization (DMM) model (all groups *n*=4–6). (A–D and I–L) sham mice; (E–H and M–P) DMM mice; time points were (A, E, I, M) 2 weeks, (B, F, J, N) 4 weeks, (C, G, K, O) 8 weeks, and (D, H, L, P) 12 weeks after surgery. CLCA1-positive synovial lining cells (A–H) and chondrocytes (I–P) were detected at all time points at similar numbers with similar staining intensities as revealed by immunohistochemistry using anti-murine CLCA antibody α-m3-C-1p with diaminobenzidine as chromogen (brown) and hematoxylin counterstain (blue). Scale bars = 50 µm (shown in H for A–H and in P for I–P).

Due to their crucial role of synovial lining cells in a wide spectrum of articular joint diseases, we further investigated possible changes in CLCA1 expression in two models of arthritis. First, we examined immunohistochemical staining patterns in murine knee joints in a commonly used DMM model of osteoarthritis.^42^ Induction of DMM-associated pathological lesions, including structural changes, inflammatory responses, and synovial cell proliferation, was verified by histological scoring following established OARSI and synovitis scoring schemes for each mouse (Supplementary Table S2).^32,33^ Staining for the CLCA1 protein was rather constant and continuous throughout the synovial lining cells and chondrocytes of DMM-treated mice at 2, 4, 8, and 12 weeks after surgery when compared with sham-treated mice at the same time points (*n*=4–6), respectively (Fig. 5).

**Figure 6.**
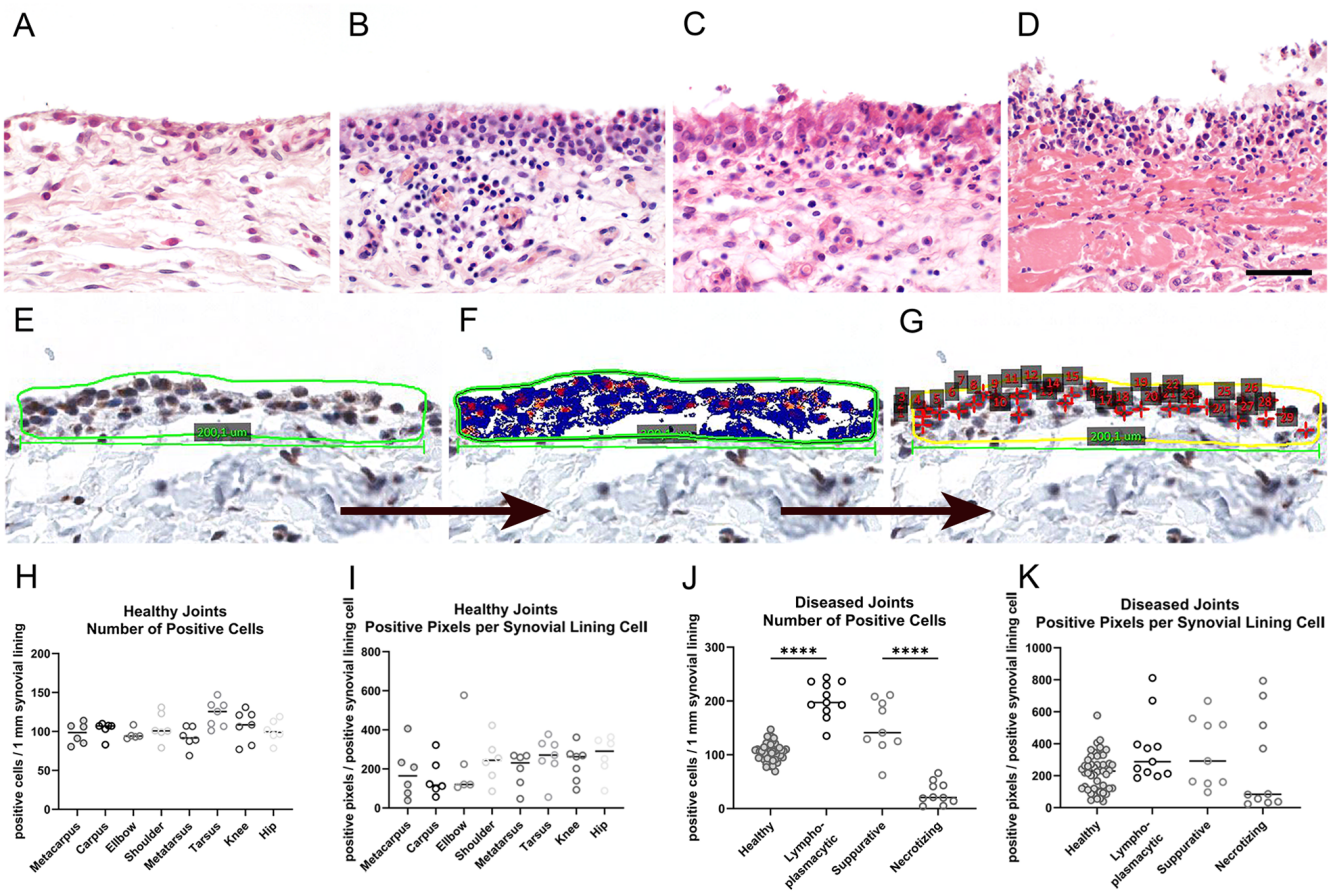
Expression of CLCA1 in articular synoviocytes in porcine osteoarthritis. (A–D) Hematoxylin and eosin–stained histological sections of examples of synovium from porcine knee joints. (A) healthy joint (*n*=11); (B) chronic lymphoplasmacytic arthritis (*n*=4); (C) suppurative arthritis with numerous infiltrating neutrophils (*n*=6); (D) necrotizing arthritis with damage and loss of synoviocytes (*n*=9); (E–G) workflow of digital analysis of immunohistochemically stained synovium using the anti-porcine CLCA1 antibody p1-N-1ab-p with diaminobenzidine as chromogen (brown) and hematoxylin counterstain (blue). (E) Annotated region of interest; (F) signal identification and pixel count using the v9 algorithm (blue color highlighting nuclei and negative areas; red and orange color highlighting the positively stained areas). (G) calculation of positive cells per region of interest. Scale bar = 50 µm (A–G). (H) Number of CLCA1-expressing cells per mm of synovial lining in various healthy porcine joints; (I) numbers of positive pixels indicative of CLCA1 expression per positive synovial lining cell in different healthy articular joints; (J) numbers of CLCA1-expressing cells per mm of synovial lining in healthy joints compared with diseased joints; (K) numbers of positive pixels indicative of CLCA1 expression per positive synovial lining cell in healthy and diseased porcine joints. Data are shown as scatter plots highlighting the median. ****p<0.0001.

Next, we examined synovium from 39 diarthrodial joints from 19 farm pigs suffering from spontaneous arthritis and compared them with 76 healthy joints from 11 farm pigs submitted for routine necropsy to our diagnostic veterinary pathology service. Synovial tissues were histologically classified into four categories, including healthy (76 joints of 11 animals; Fig. 6A), chronic lymphoplasmacytic (16 joints of 4 animals, Fig. 6B), suppurative (13 joints of 6 animals, Fig. 6C), and necrotizing arthritis (10 joints of 9 animals, Fig. 6D). Following established workflows, five randomly distributed areas of 200 µm in length ±0.5 µm were annotated (Fig. 6E), accounting for 1 mm length in total. The number of CLCA1-labeled synovial lining cells was manually counted (Fig. 6F), and positive pixels were calculated utilizing the pixel count V9 algorithm (Leica; Fig. 6G). If one anatomic location was affected on both (left and right) sides, the mean was determined. We found that both the number of CLCA1-expressing cells per 1-mm synovial lining (Fig. 6H) and the number of CLCA1-positive pixels per synovial lining cell (Fig. 6I) were similar among the healthy joints from different anatomical locations. In contrast, the number of CLCA1-expressing synoviocytes per 1-mm synovial lining significantly varied between the four groups of healthy and diseased joints (Fig. 6J). Specifically, the number of CLCA1-expressing synoviocytes more than doubled in the chronic lymphoplasmacytic synovitis with highly proliferated synovial lining, whereas it increased only slightly under conditions of more acute suppurative inflammation. In sharp contrast, only few CLCA1-expressing synoviocytes remained in pigs with necrotizing arthritis, characterized by drastic degeneration and loss of the synovial lining. However, the number of CLCA1-positive pixels per synovial lining cell remained rather constant between the healthy joints and lymphoplasmacytic and suppurative synovitis, consistent with more or less unchanged CLCA1 protein content per cell (Fig. 6K). The mean intensity of CLCA1 staining per cell appeared reduced only in joints with necrotizing arthritis with severely damaged synoviocytes, however, with large variations between the number of CLCA1-expressing cells between individual joints and animals (Fig. 6K).

### CLCA1 Expression in a Porcine Model of Early Post-traumatic Osteoarthritis

**Figure 7.**
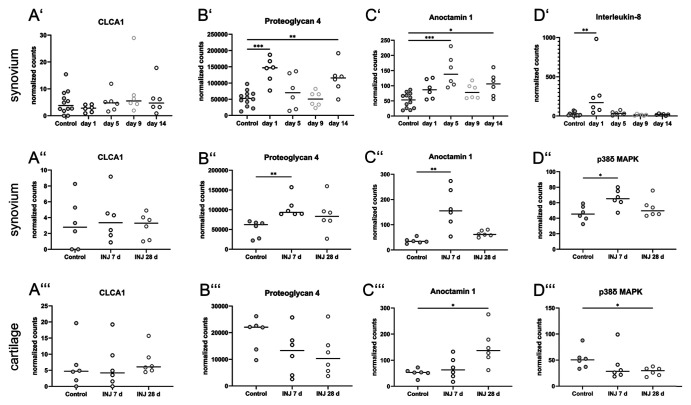
Transcriptome data analyses of synovium (A’–D’) and cartilage samples (A’’–D’’’) from three previous studies of a porcine early-osteoarthritis (OA) model (*n*=6).^43,44^ The data were retrieved from the Sequence Read Archive (SRA) database (accession numbers ERP009122, ERP107920, ERP024317). The first study focussed on synovium at early time points from day 1 to 14 after surgery (A’–D’), whereas the second and third studies focussed on the synovium (A’’–D’’) or the cartilage (A’’’–D’’’), respectively, on days 7 and 28. (A) Expression levels of CLCA1 and (B–D) other select target genes in surgically treated (INJ) pigs during the course of the experiment are depicted in comparison with sham-operated control groups. (A’–A’’’) Constant basal expression of CLCA1 was detected in the synovium and cartilage in all three studies of early OA. (B’–B’’’) Expression levels of proteoglycan 4 as major articular lubricant increased significantly after (B’) 24 hr and (B’’) 7 days in the synovium but (B’’’) seemed slightly reduced in the cartilage during the early course of disease. (C’–C’’) Anoctamin-1 had significantly higher expression levels on days 5 (C’) and 7 (C’’) in the synovium. (C’’’) In the cartilage, however, significant changes were detected only after 28 days. (D’) Expression of interleukin-8, a pro-inflammatory cytokine, significantly increased after 24 hr but not at later time points. (D’’) p38δ MAPK showed a significant increase in the synovium after 7 days, whereas no differences in expression levels were detected after 28 days. (D’’’) In contrast, p38δ MAPK had significantly increased expression levels in the cartilage albeit only after 28 days. Data are shown as scatter plots highlighting the median. *p<0.05, **p<0.01, ***p<0.001.

Finally, we asked whether publicly accessible transcriptome data sets from relevant animal models could be used to further elucidate the role of CLCA1 in joint disease. To this end, we analyzed data from two previous experiments that had employed porcine models of early post-traumatic osteoarthritis. First, Sieker and coworkers from the Boston Children’s Hospital, Harvard Medical School, had conducted anterior cruciate ligament transection (ACLT) in Yucatan minipigs [*n*=6 per time point; NCBI Sequence Read Archive (SRA) database accession number ERP009122].^43^ Our analyses of the available transcriptome data revealed that CLCA1 mRNA was expressed in the synovium rather constantly on a basal level during the very early course of disease until day 14 after ACLT (Fig. 7A’).

Second, we analyzed transcriptome data from a different experimental approach by looking at changes in gene expression levels both in the synovium and cartilage of pigs 7 and 28 days after surgical ACLT or sham treatment (NCBI SRA database accession numbers ERP107920; ERP024317). The authors of that study described an increase in synovial tissue area, intimal thickness, and stromal cellularity by histology for both time points with more pronounced changes after 28 days.^43^ In the cartilage, loss of glycosaminoglycans and penetration of the tidemark by vasculature was observed at both time points, whereas increased chondrocyte density and chondrocyte clusters were only observed after 28 days.^44^ Here, we again identified rather constant CLCA1 mRNA expression both in the synovium (Fig. 7A’ and 7A”) and cartilage (Fig. 7A’’’).

We also examined mRNA levels of potential interactors of CLCA1 which we speculated on the basis of interactions and pathways that had previously been established for CLCA1 in intestinal and respiratory mucosal membranes.^13–15,18,45–47^ For example, normalized counts of proteoglycan 4 mRNA were significantly elevated during the early phase of ACLT in the synovium (Fig. 7B’ and 7B’’). In the cartilage, however, proteoglycan 4 levels appeared slightly reduced on days 7 and 28 (Fig. 7B’’’). Normalized counts of anoctamin-1 (ANO1) mRNA, a calcium-activated chloride channel that interacts with CLCA1,^45^ significantly increased reaching their maximum on day 5 in the first study (Fig. 7C’). These results matched the data obtained from the second study with most pronounced expressional ANO1 increase on day 7 (Fig. 7C”). In the cartilage, however, significantly increased expression was observed only on day 28 (Fig. 7C’’’). Furthermore, IL-8 mRNA expression increased significantly in the synovium after ACTL only on the first day after surgical intervention and then returned to baseline expression (Fig. 7D’). As another possible interactor with CLCA1, p38δ MAPK,^18^ like ANO1, had significantly increased mRNA levels at day 7 in the synovium (Fig. 7D”), but was reduced at both experimental time points in the cartilage (Fig. 7D’’’).

Surprisingly, mRNA levels of aggrecan as one of the major extracellular matrix components remained unchanged at all time points analyzed when compared with the sham-treated pigs (Supplementary Fig. S4).

## Discussion

Despite more than 25 years of research on CLCA1 since its discovery,^1^ its role in health and disease is far from understood. The only common denominator so far has been the firmly established notion that across all mammalian species investigated to date, CLCA1 is exclusively expressed in goblet and other mucus-producing cells and glands, foremost in intestinal, respiratory, and urogenital mucous membranes.^3–5,23^ Here, we expand this paradigm by identifying consistent expression of CLCA1 by synovial lining cells and chondrocytes in diarthrodial joints.

The articular expression pattern of CLCA1 seems conserved among mammalian species and different diarthrodial joints investigated so far. Cartilage in other locations, such as the ear, trachea, and nasal septum, seems not to express CLCA1. Interestingly, murine joints started to express CLCA1 only during the first weeks of life around onset of regular joint usage, followed by continuous expression even in degenerate joints of aged animals. Fibroblast-like synoviocytes constitute approximately 80% of the synovial lining cells in normal murine synovium, while roughly 20% usually represent macrophage like synoviocytes.^48^ Proteoglycan 4, also known as lubricin, is a mucin-like glycoprotein predominantly expressed in fibroblast-like, or type B, synoviocytes as well as superficial and, to a lesser extent, intermediate zone chondrocytes.^49–52^ It is essential for proper lubrication and frictionless motion of articular joints.^53,54^ Our observations on its widely overlapping cellular expression pattern with that of proteoglycan 4 and lack of CLCA1 expression in CD68-positive (probably type A) synoviocytes suggest that it is likely expressed by fibroblast-like synoviocytes and superficial to intermediate zone chondrocytes. This implies obvious parallels to CLCA1 expression in mucous membranes and glands that also express one or several of the different mucin glycoproteins.^3–5,23^ In light of recent structural and experimental evidence toward its function in cleaving glycosylated substrates and altering mucin architecture,^17,46^ we speculate that similar interactions may exist between CLCA1 with mucins or mucin-like glycoproteins on mucous membranes and with components of the synovial fluid in diarthrodial joints.

Incidentally, CLCA1 had previously been found as a constituent of the synovial fluid in osteoarthritic horses without differences between diseased and healthy joints.^55^ Together with our findings on its cellular expression pattern and its established structure as a canonically secreted glycoprotein, this implies its secretion by synoviocytes and possibly chondrocytes, similar to its established secretion by goblet cells in mucous membranes.^1,3–5^ Finally, we established rather constant articular expression of CLCA1 under various conditions of joint diseases, including murine and porcine models of osteoarthritis and spontaneous arthritis in farm pigs. Overall, our discovery of articular expression of CLCA1 together with many obvious similarities to its secretion by mucous membranes of the intestine, lung, and other organs raises new questions toward its functional roles in this microenvironment in health and disease.

Joint diseases are among the leading causes of mobility impairment in aged people, including conditions such as osteoarthritis and rheumatoid arthritis. Here, the interaction between cartilage and synovium has caught increasing attention during the past years.^56–58^ Identification of CLCA1 in both compartments therefore may add an interesting component to the understanding of this interaction. However, a recent report on CLCA1-deficient pigs failed to mention any evidence of articular pathology that could have contributed to the understanding of its role in this context.^19^ On the other hand, CLCA1-deficient mice had slightly but significantly reduced swelling of the joints as well as decreased inflammation and destruction scores in an acute antigen-induced arthritis model.^30^ Still, the authors attributed this to dysfunctional effects in dorsal root ganglia while synovial and chondrocytic expression of CLCA1 was unknown at that time.

Clues toward CLCA1’s actual function in joints may possibly be deduced from its established roles and interaction partners in other tissues. For example, the amino-terminus of CLCA1 interacts with the extracellular domain of the calcium-activated chloride channel anoctamin-1 (ANO1, also known as TMEM16A) to stimulate chloride ion (Cl^−^) movement.^13,14,59^ Furthermore, CLCA1 stimulates Cl^−^ current via activation of ANO1 with concurrent changes in protein synthesis, thereby largely contributing to kidney injury in aging mice.^60^ Volume regulation in rat articular chondrocytes relies on expression of ANO1 that is at least partially responsible for the swelling-activated Cl^−^ currents, consistent with a role in volume regulation of chondrocytes during hypoosmotic conditions.^61^ A selective blocker of the Ca^2+^-activated chloride channel and ANO1, (CaCC_inh_)-A01, appeared most effective in preventing swelling in rabbit osteoarthritic chondrocytes.^62^ In our study, we found significantly elevated ANO1 mRNA copy numbers in the synovium and chondrocytes at 7 and 28 days after surgery, respectively, in a porcine model of early post-traumatic osteoarthritis. Likewise, ANO1 was increasingly expressed in a subset of patients with knee osteoarthritis with altered patterns of matrix protein expression in humans.^63^ Clearly, the established interaction of CLCA1 with ANO1 in other tissues warrants a closer look at a similar scenario in joints with possible contributions to matrix hydration, control of chondrocyte volume, and homeostasis of synovial fluid.

Another interesting aspect is the established role of CLCA1 in the IL-13/p38δ MAPK/mucus overproduction signaling pathway. High levels of IL-13 in the lungs of human patients with chronic obstructive pneumonia are thought to promote CLCA1 signaling, resulting in activation of p38δ MAPK and elevated mucus levels, especially of MUC5AC.^18^ Of note, IL-13 is also consistently expressed in rheumatoid synovium and p38δ MAPK is highly expressed in synovial tissue from rheumatoid arthritis patients.^64,65^ Moreover, p38γ/δ-deficient mice failed to develop collagen-induced arthritis, bone destruction, and joint damage.^66^ Obviously, the role of CLCA1 in the IL-13/p38δ MAPK signaling pathway in joint disorders needs further clarification.

Finally, strong and repeated evidence of CLCA1 being involved in early immune functions, such as pro-inflammatory activation of a subset of macrophages, raises the question of similar interactions in articular microenvironments. For example, CLCA1 is involved in the upregulation of IL-17 and CXCL-1, the murine orthologue to human CXCL-8 also known as IL-8, with downstream effects on leukocyte recruitment to inflamed tissue.^20^ Interestingly, IL-17 also promotes rheumatoid arthritis and CXCL-8 is among the major pro-inflammatory cytokines in human osteoarthritis where it is thought to aggravate disease progression.^67–72^ CXCL-8 together with CXCL-11 also suppresses chondrocyte proliferation and promotes apoptosis in cultured chondrocytes from patients with osteoarthritis.^70^ Still, expression levels of CLCA1 did not differ between infected and uninfected mouse lungs,^20^ which is reminiscent of the seemingly constant expression of CLCA1 in healthy and inflamed joints as observed in our study. Whether this may point toward regulatory interaction on the protein level or other post-transcriptional mechanisms remains to be established.

Taken together, the identification of CLCA1 expression in synoviocytes and articular chondrocytes sets the stage for further studies on possible roles of this multifunctional glycoprotein in osteoarthritis and other joint diseases. Several parallels are conceivable to previous work on CLCA1 being secreted by goblet cells into the mucus of a wide spectrum of mucous membranes. These warrant further investigations toward similar contributions to articular anion conductance, proteoglycan homeostasis, and inflammation, all of which have been established for CLCA1 only in the respiratory and intestinal microenvironments so far.

## Supplemental Material

sj-pdf-1-jhc-10.1369_00221554261423720 – Supplemental material for The Chloride Channel Regulator, Calcium-Activated-1 Is Expressed in Synoviocytes and Articular Chondrocytes in Health and Disease Supplemental material, sj-pdf-1-jhc-10.1369_00221554261423720 for The Chloride Channel Regulator, Calcium-Activated-1 Is Expressed in Synoviocytes and Articular Chondrocytes in Health and Disease by Judith Bushe, Jenny Fürstenau, Florian Bartenschlager, Simon Dökel, Vladimir M. Jovanovic, Gundula Rösch, Zsuzsa Jenei-Lanzl, Frank Zaucke, Kristina Dietert and Achim D. Gruber in Journal of Histochemistry & Cytochemistry

sj-pdf-2-jhc-10.1369_00221554261423720 – Supplemental material for The Chloride Channel Regulator, Calcium-Activated-1 Is Expressed in Synoviocytes and Articular Chondrocytes in Health and Disease Supplemental material, sj-pdf-2-jhc-10.1369_00221554261423720 for The Chloride Channel Regulator, Calcium-Activated-1 Is Expressed in Synoviocytes and Articular Chondrocytes in Health and Disease by Judith Bushe, Jenny Fürstenau, Florian Bartenschlager, Simon Dökel, Vladimir M. Jovanovic, Gundula Rösch, Zsuzsa Jenei-Lanzl, Frank Zaucke, Kristina Dietert and Achim D. Gruber in Journal of Histochemistry & Cytochemistry

sj-pdf-3-jhc-10.1369_00221554261423720 – Supplemental material for The Chloride Channel Regulator, Calcium-Activated-1 Is Expressed in Synoviocytes and Articular Chondrocytes in Health and Disease Supplemental material, sj-pdf-3-jhc-10.1369_00221554261423720 for The Chloride Channel Regulator, Calcium-Activated-1 Is Expressed in Synoviocytes and Articular Chondrocytes in Health and Disease by Judith Bushe, Jenny Fürstenau, Florian Bartenschlager, Simon Dökel, Vladimir M. Jovanovic, Gundula Rösch, Zsuzsa Jenei-Lanzl, Frank Zaucke, Kristina Dietert and Achim D. Gruber in Journal of Histochemistry & Cytochemistry
