# Supplementary material for: The Chloride Channel Regulator, Calcium-Activated-1 Is Expressed in Synoviocytes and Articular Chondrocytes in Health and Disease
Source: J Histochem Cytochem. 2026 Mar 15:00221554261423720. Online ahead of print. doi: 10.1369/00221554261423720 (PMC12989442; doi:10.1369/00221554261423720)
Supplement: sj-pdf-1-jhc-10.1369_00221554261423720 – Supplemental material for The Chloride Channel Regulator, Calcium-Activated-1 Is Expressed in Synoviocytes and Articular Chondrocytes in Health and Disease [file sj-pdf-1-jhc-10.1369_00221554261423720.pdf]

Supplemental Table S1 sheet 2

Animal ID	Animal no.	joint	condition
V1811_19	1	MCL	1
V1811_19	1	CL	1
V1811_19	1	EL	1
V1811_19	1	SL	1
V1811_19	1	MCR	1
V1811_19	1	CR	1
V1811_19	1	ER	1
V1811_19	1	SR	1
V1811_19	1	MTL	1
V1811_19	1	TL	1
V1811_19	1	KL	1
V1811_19	1	HL	1
V1811_19	1	MTR	1
V1811_19	1	TR	1
V1811_19	1	KR	1
V1811_19	1	HR	1
V1812_19	2	MCL	1
V1812_19	2	CL	1
V1812_19	2	EL	1
V1812_19	2	MCR	1
V1812_19	2	CR	1
V1812_19	2	ER	1
V1812_19	2	SR	1
V1812_19	2	MTL	1
V1812_19	2	TL	1
V1812_19	2	KL	1
V1812_19	2	HL	1
V1812_19	2	MTR	1
V1812_19	2	TR	1
V1812_19	2	KR	1
V1812_19	2	HR	1
V1813_19	3	MCL	1
V1813_19	3	CL	1
V1813_19	3	EL	1
V1813_19	3	SL	1
V1813_19	3	CR	1
V1813_19	3	ER	1
V1813_19	3	SR	1
V1813_19	3	MTL	1
V1813_19	3	TL	1
V1813_19	3	KL	1
V1813_19	3	MTR	1
V1813_19	3	TR	1
V1813_19	3	KR	1
V1813_19	3	HR	1
S201_16	7	MLC	1
S201_16	7	KL	1
S201_16	7	EL	1

abbrevation	condition
1	healthy
2	lymphoplasmacellular
3	suppurative
4	necrotizing

abbrevation	joint
MCL	left metacarpus
MCR	right metacarpus
CL	left carpus
CR	right carpus
EL	left ellbow
ER	right ellbow
SL	left shoulder
SR	right shoulder
MTL	left metatarsus
MTR	right metatarsus
TL	left tarsus
TR	right tarsus
KL	left knee
KR	right knee
HL	left hip
HR	right hip

S37_17	10	SL	1
S37_17	10	TL	1
S35_20	17	CL	1
S58_17	21	TL	1
S58_17	21	HL	1
S58_17	21	MTR	1
S58_17	21	TR	1
S58_17	21	HR	1
S59_17	22	MTL	1
S59_17	22	TL	1
S59_17	22	KL	1
S59_17	22	HL	1
S59_17	22	MTR	1
S59_17	22	TR	1
S59_17	22	KR	1
S59_17	22	HR	1
S387_16	23	SR	1
S254_16	24	MCL	1
S254_16	24	CL	1
S254_16	24	EL	1
S254_16	24	SL	1
S254_16	24	MTL	1
S254_16	24	TL	1
S254_16	24	KL	1
S254_16	24	HL	1
S895_19	25	MCL	1
S895_19	25	CL	1
S895_19	25	KL	1
S212_16	5	CR	2
S152_18	9	TL	2
S152_18	9	KL	2
S152_18	9	TR	2
S152_18	9	KR	2
S387_16	23	EL	2
S387_16	23	SL	2
S387_16	23	CR	2
S387_16	23	MTL	2
S387_16	23	TL	2
S387_16	23	KL	2
S387_16	23	MTR	2
S387_16	23	TR	2
S387_16	23	KR	2
S387_16	23	HR	2
S895_19	25	TL	2
S228_16	6	CL	3
S437_16	8	EL	3
S437_16	8	SL	3
S437_16	8	ER	3
S437_16	8	SR	3
S437_16	8	TL	3
S437_16	8	KL	3
S437_16	8	TR	3

S437_16	8	KR	3
S345_19	15	EL	3
S36_20	18	TR	3
S37_20	19	TL	3
S211_16	4	HR	4
S212_16	5	TR	4
S18_18	11	TL	4
S18_18	11	KL	4
S23_18	12	HR	4
S98_18	13	TR	4
S99_18	14	EL	4
S34_20	16	CL	4
S35_20	17	TL	4
S37_20	19	KL	4
S37_17	10	KL	1
S387_16	23	MLC	1 - 2
S387_16	23	CL	1 - 2
S387_16	23	ER	1 - 2
S201_16	7	CL	3
S189_20	20	TL	3 -4
