# Supplementary material for: The Chloride Channel Regulator, Calcium-Activated-1 Is Expressed in Synoviocytes and Articular Chondrocytes in Health and Disease
Source: J Histochem Cytochem. 2026 Mar 15:00221554261423720. Online ahead of print. doi: 10.1369/00221554261423720 (PMC12989442; doi:10.1369/00221554261423720)
Supplement: sj-pdf-2-jhc-10.1369_00221554261423720 – Supplemental material for The Chloride Channel Regulator, Calcium-Activated-1 Is Expressed in Synoviocytes and Articular Chondrocytes in Health and Disease [file sj-pdf-2-jhc-10.1369_00221554261423720.pdf]

Supplemental Table S1

Animals and tissue samples

abbrevations:

ID	identification number
DMM	Destabilization of the Medial Meniscus
sham	sham operated control group

Murine Samples

Healthy mice for expression analysis

Number	age	gender	samples
V245/17	12 weeks	male	major diathrodial joints
V246/17	12 weeks	male	major diathrodial joints
V247/17	12 weeks	male	major diathrodial joints
V348/17	12 weeks	female	major diathrodial joints
V 353/17	12 weeks	male	major diathrodial joints
V 354/17	12 weeks	male	major diathrodial joints
V 355/17	12 weeks	male	major diathrodial joints
V 356/17	12 weeks	female	major diathrodial joints
V 357/17	12 weeks	female	major diathrodial joints
V 358/17	12 weeks	female	major diathrodial joints

Age comparisons

emryonal stages E12.5, E16.5, E 18.5 and postpartum day 1 n=3

Animal ID	age	gender	samples
E12.5	12.5 days of pregnancy	n=3 both genders	whole body
E16.5	16.5 days of pregnancy	n=3 both genders	whole body
E18.5	18.5 days of pregnancy	n=3 both genders	whole body
p1	1 day	n=3 both genders	whole body
p10 1	10 days	male	hindlimb
p10 2	10 days	female	hindlimb
p10 3	10 days	female	hindlimb
p20 1	20 days	male	hindlimb
p20 2	20 days	male	hindlimb
p20 3	20 days	male	hindlimb
p30 1	30 days	female	hindlimb
p30 2	30 days	female	hindlimb
p30 3	30 days	female	hindlimb
V1042/15	8 W	female	hindlimb
V1044/15	8 W	female	hindlimb
V1178/15	8 W	female	hindlimb
V1179/15	8 W	male	hindlimb
V1180/15	8 W	male	hindlimb
V1183/15	8 W	male	hindlimb
V642/15	6 M	female	hindlimb
V643/15	6 M	female	hindlimb
V1235/15	6 M	female	hindlimb
V1298/15	6 M	female	hindlimb
V267/16	6 M	female	hindlimb
V268/16	6 M	female	hindlimb
V270/16	6 M	female	hindlimb
V1240/15	12 M	female	hindlimb
V1242/15	12 M	female	hindlimb
V678/16	12 M	female	hindlimb
V679/16	12 M	female	hindlimb
V680/16	12 M	female	hindlimb
V335/16	18 M	female	hindlimb
V336/16	18 M	female	hindlimb
V337/16	18 M	female	hindlimb
V338/16	18 M	female	hindlimb

Additional mice for age and gender comparison

Animal ID	age	gender	samples
ZA1	3 months	female	knee
ZA2	3 months	female	knee
ZA3	3 months	female	knee
ZA4	3 months	male	knee
ZA5	3 months	male	knee
ZA6	3 months	male	knee
ZA7	6 months	female	knee
ZA8	6 months	female	knee
ZA9	6 months	female	knee
ZA10	6 months	male	knee
ZA11	6 months	male	knee
ZA12	6 months	male	knee
ZA13	18 months	female	knee
ZA14	18 months	female	knee
ZA15	18 months	female	knee
ZA16	18 months	male	knee
ZA17	18 months	male	knee
ZA18	18 months	male	knee

DMM-Model

Animal ID	Time point after surgery (weeks)	Surgery
199	2 weeks	sham
200	2 weeks	sham
201	2 weeks	sham
202	2 weeks	sham
203	2 weeks	sham
209	2 weeks	sham
204	2 weeks	DMM
205	2 weeks	DMM
206	2 weeks	DMM
229	2 weeks	DMM
232	2 weeks	DMM
27	4 weeks	sham
28	4 weeks	sham
29	4 weeks	sham
31	4 weeks	sham
32	4 weeks	sham
33	4 weeks	sham
16	4 weeks	DMM
17	4 weeks	DMM
18	4 weeks	DMM
23	4 weeks	DMM
24	4 weeks	DMM
91	8 weeks	sham
93	8 weeks	sham
94	8 weeks	sham
95	8 weeks	sham
98	8 weeks	sham
80	8 weeks	DMM
82	8 weeks	DMM
86	8 weeks	DMM
88	8 weeks	DMM
89	8 weeks	DMM
131	12 weeks	sham
133	12 weeks	sham
134	12 weeks	sham
143	12 weeks	sham
145	12 weeks	sham
137	12 weeks	DMM
138	12 weeks	DMM

139	12 weeks	DMM
140	12 weeks	DMM
141	12 weeks	DMM
163	12 weeks	DMM

Additional mice for investigation of non-articular cartilage

Animal ID	age	gender	samples
Z1	12 weeks	male	ear, nose, trachea
Z2	12 weeks	female	ear, nose, trachea
Z3	14 weeks	male	ear, nose, trachea
Z4	14 weeks	female	ear, nose, trachea
Z5	16 weeks	male	ear, nose, trachea
Z6	16 weeks	male	ear, nose, trachea
V378/23	12 weeks	female	trachea
V381/23	12 weeks	female	trachea
V382/23	12 weeks	female	trachea

Porcine Samples

Healthy pigs for expressional analyses

Animal ID	age	gender	samples	additional samples
V1811/19	3 months	female	major diarthrodial joints cartilage and synovium	trachea, ear, nose
V1812/19	4 months	female	major diarthrodial joints cartilage and synovium	trachea, ear, nose
V1813/19	3 months	male	major diarthrodial joints cartilage and synovium	trachea, ear, nose
S513/25	4 months	male	-	trachea, ear, nose
S521/25	3 months	male	-	trachea, ear, nose

Healthy and diseased pigs for digital analysis

detailed information regarding sampled synovium and condition of the joint in sheet 2

Number	age	gender
S201/16	3 weeks	male
S211/16	4 weeks	female
S212/16	6 weeks	male
S228/16	3 months	male
S254/16	8 weeks	female
S387/16	2 years	female
S437/16	3 months	male
S37/17	4 weeks	female
S58/17	6 months	male
S59/17	5 months	female
S018/18	7 weeks	female
S022/18	4 months	female
S023/18	5 months	female
S098/18	3 weeks	male
S099/18	3 weeks	female
S152/18	3 months	male
S044/19	7 weeks	male
S345/19	2 months	male
S895/19	3 months	female
V1811/19	3 months	female
V1812/19	4 months	female
V1813/19	3 months	female
S034/20	8 weeks	female
S035/20	8 weeks	male
S036/20	8 weeks	female
S037/20	8 weeks	female
S189/20	6 weeks	male

Equine samples

Animal ID	age	gender	samples	breed
S445/18	15 Jahre	male castrated	shoulder	Shetlandpony
S352/15	13 Jahre	male castrated	metacarpophalangeal joint, hoof joint, krongelenk	German Warmblood
S358/15	11 years	male castrated	hoof, krongelenk, metacarpophalangeal joint	Irish Sporthorse
S362/15	12 years	male castrated	shoulder, knee	Warmblood
S446/15	2 years	male castrated	shoulder, knee	German Warmblood
S615/15	7 years	male non	tarsal joint, knee	Friesian
S41/17	8 months	male	tarsal joint, knee	German Warmblood
S145/19	6 weeks	female	tarsal joints	German Warmblood
S475/19	3 months	female	carpal, knee, hip	Friesian
S373/19	7 weeks	female	carpal, shoulder	German Warmblood
S372/19	6 weeks	male	shoulder, tarsus	German Warmblood

Human Samples

Number	age	gender	samples	condition
17	83 years	male	Synovium knee and tarsal joint	healthy
19	78 years	male	Synovium knee and tarsal joint	healthy
21	66 years	male	Synovium knee and tarsal joint	healthy
22	95 years	female	Synovium knee and tarsal joint	healthy
23	74 years	male	Synovium knee and tarsal joint	healthy
24	94 years	female	Synovium knee and tarsal joint	healthy
25	69 years	male	Synovium knee and tarsal joint	healthy
26	97 years	female	Synovium knee and tarsal joint	healthy
27	92 years	male	Synovium knee and tarsal joint	healthy
28	95 years	female	Synovium knee and tarsal joint	healthy
