# Supplementary material for: The Chloride Channel Regulator, Calcium-Activated-1 Is Expressed in Synoviocytes and Articular Chondrocytes in Health and Disease
Source: J Histochem Cytochem. 2026 Mar 15:00221554261423720. Online ahead of print. doi: 10.1369/00221554261423720 (PMC12989442; doi:10.1369/00221554261423720)
Supplement: sj-pdf-3-jhc-10.1369_00221554261423720 – Supplemental material for The Chloride Channel Regulator, Calcium-Activated-1 Is Expressed in Synoviocytes and Articular Chondrocytes in Health and Disease [file sj-pdf-3-jhc-10.1369_00221554261423720.pdf]

Supplemental Material

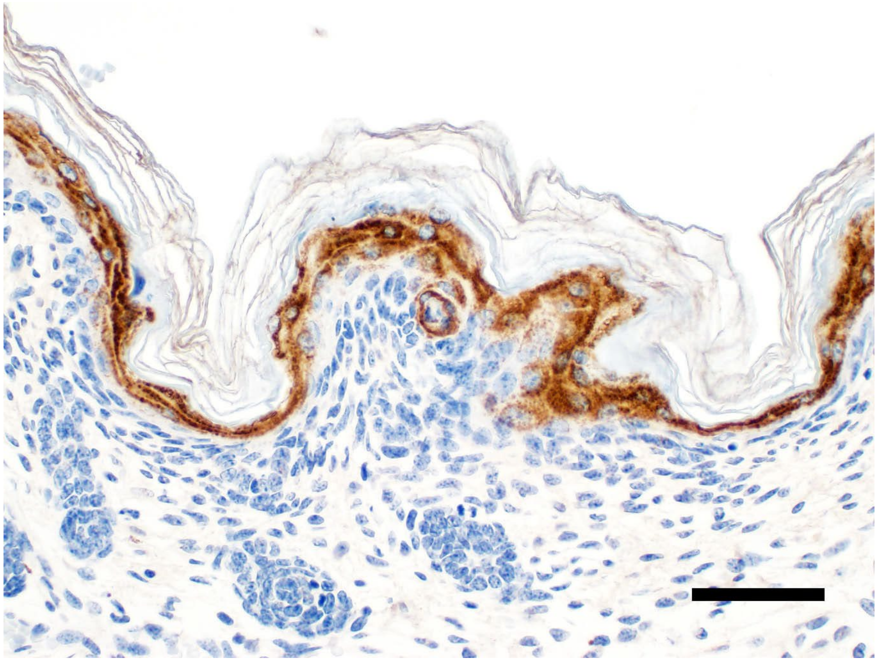

Figure S1. Positive control for the immunohistochemical detection of the CLCA2 protein in the murine skin (n=3) using anti-murine CLCA2-antibody m5-C1-a with diaminobenzidine as chromogen (brown) and hematoxylin counterstain (blue).[35] Strong signals for CLCA2 were detected in cornifying keratinocytes only. Scale bar = 100 µm.

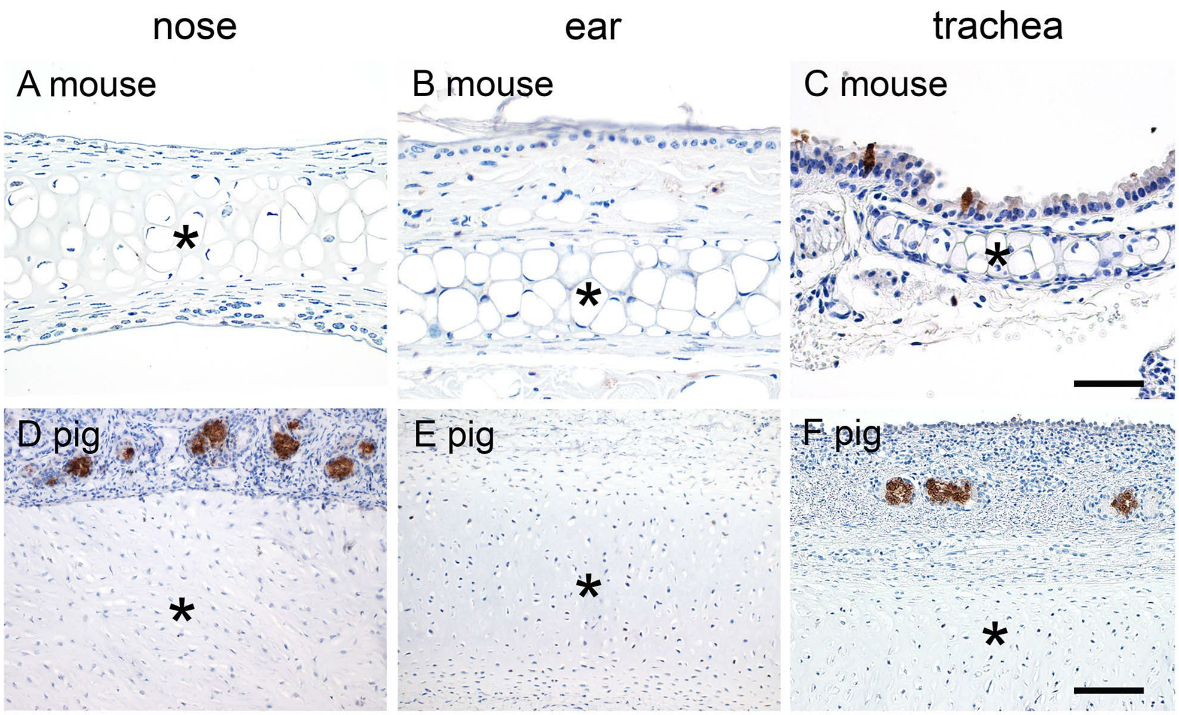

Figure S2. Lack of CLCA1 expression in non-articular hyaline cartilage. Murine (A-C) and porcine (D-F) nasal septal (A, D), auricular (B, E) and tracheal (C, F) tissues (n=3) were immunohistochemically stained with anti-murine CLCA1 antibody α-m3-C-1p (A-C) or anti-porcine CLCA1 antibody P1-N-ab-P (D-F), using diaminobenzidine as chromogen (brown) and hematoxylin counterstain (blue). No CLCA1 expression was detected in any of the hyaline cartilage structures examined (asterisks), while strong CLCA1 expression was detected (brown signals) in adjacent murine tracheal goblet cells (C) and porcine submucosal mucinous glands in the nose (D) and trachea (F). Bar = 100 µm (A-C) or 200 µm (D-F).

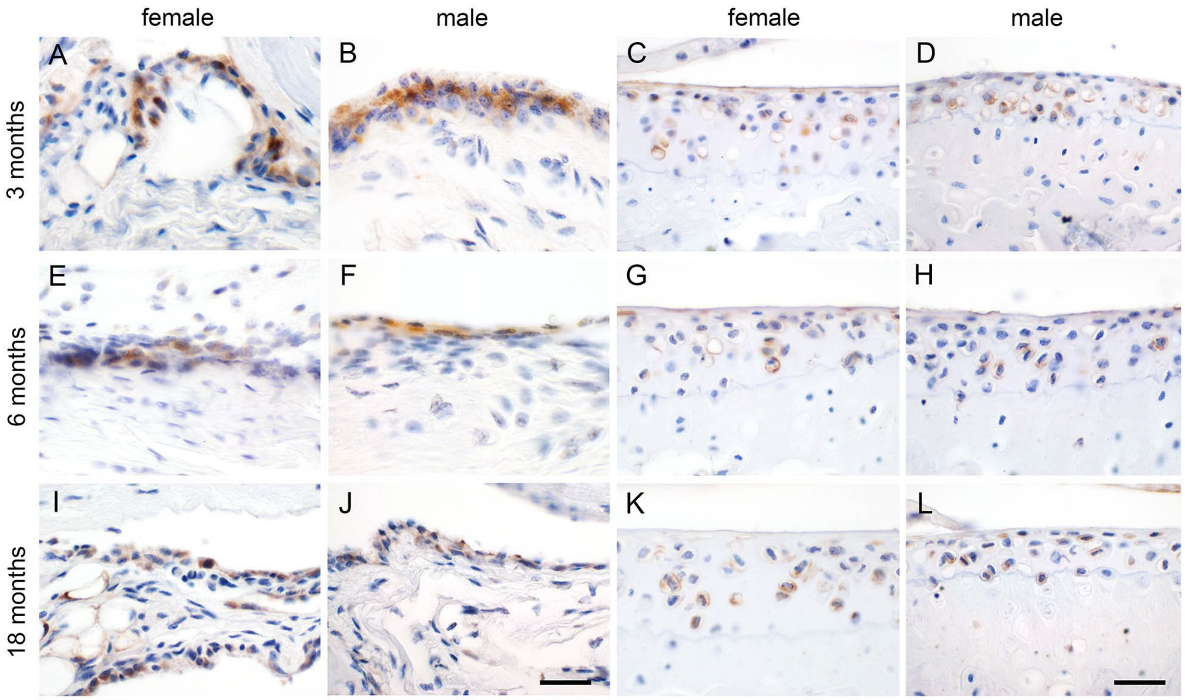

Figure S3. Sex- and age-dependent expression of CLCA1 protein in synoviocytes and chondrocytes of murine knee joints. Immunohistochemical staining using antibody α-m3-C-1p, diaminobenzidine as chromogen (brown) and hematoxylin counterstain (blue). Both female and male mice appeared to express slightly less CLCA1 in the synovium (left two columns) and in the cartilage (right two columns) with increasing age, here ranging from 3 (A-D) over 6 (E-H) to 18 months of (I-L). Scale bars = 50 µm (synovium, left half) and 70 µm (cartilage, right half).

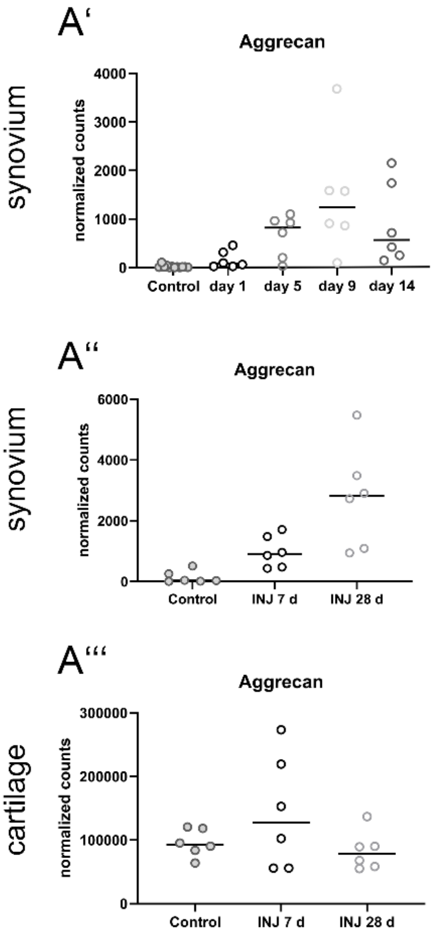

Figure S4. Expression levels of aggrecan in synovium (A', A'') and cartilage samples (A''') from three previous studies of a porcine early-osteoarthritis (OA) model (n=6).[44,45] The data were retrieved from the Sequence Read Archive (SRA) database (accession numbers ERP009122, ERP107920, ERP024317). (A'-A''') No significant changes were detected in expression levels both in the synovium at very early (A') and early (A'') time points of the experiment or in the cartilage (A''').

Table S1: See attached MS Excel file

Table S2. Pathological Outcome of Destabilization of the Medial Meniscus (DMM) Experiments in Male C57BL/6J Mice at 12 Weeks of Age (n=4-6).

Animal ID	Surgery	Time point after surgery (weeks)	OARSI score[32]	Synovitis score[33]
199_51	Sham	2	0	0
200_18	Sham	2	0	0
201_61	Sham	2	0	0
202_43	Sham	2	0	0
203_51	Sham	2	0	0
209_52	Sham	2	0	0
204_54	DMM	2	0.5	0
205_66	DMM	2	0.5	1
206_53	DMM	2	1	1
229_54	DMM	2	0.5	1
232_87	DMM	2	1	2
27_54	Sham	4	0	0
28_72	Sham	4	0.5	0
29_98	Sham	4	0	0
31_57	Sham	4	0	0
32_58	Sham	4	0	0
33_78	Sham	4	0	1
16_63	DMM	4	2	2
17_58	DMM	4	0.5	2
18_162	DMM	4	2	3
23_64	DMM	4	0.5	2
24_62	DMM	4	1	1
91_51	Sham	8	0.5	0
93_52	Sham	8	0	0
94_50	Sham	8	0	1
95_76	Sham	8	0	0
98_65	Sham	8	0	0
86_51	DMM	8	1	1
89_51	DMM	8	3	3
80_55	DMM	8	3	3

82_58	DMM	8	1	3
88_63	DMM	8	3	2
131_51	Sham	12	0	0
134_57	Sham	12	0	0
133_77	Sham	12	0	1
143_66	Sham	12	0	0
145_84	Sham	12	0.5	1
138_51	DMM	12	2	3
141_53	DMM	12	1	2
163_50	DMM	12	2	3
137_55	DMM	12	3	3
139_66	DMM	12	2	2
140_58	DMM	12	2	3

Abbreviations used: ID identification number; DMM Destabilization of the Medial Meniscus; OARSI Osteoarthritis Research Society International;
